# Supplementary figures and images for: Wheat amino acid transporters highly expressed in grain cells regulate amino acid accumulation in grain
Source: PLoS One. 2021 Feb 19;16(2):e0246763. doi: 10.1371/journal.pone.0246763 (PMC7894817; doi:10.1371/journal.pone.0246763)

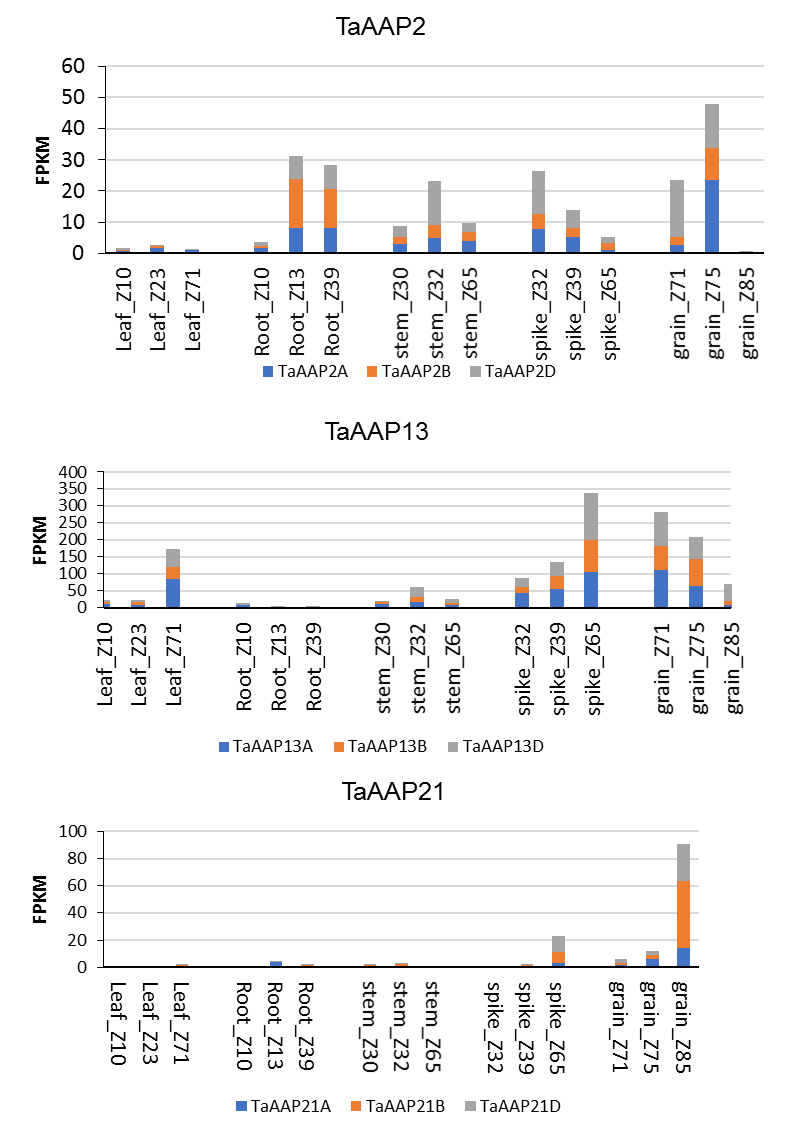

Supplement: S1 Fig — The TaAAP2, TaAAP13, and TaAAP21 expression patterns in different organs of Chinese Spring by RNA-seq at leaf, root, stem, spike and grains (Z71, 2DPA, Z75, 14DA, Z85, 30DPA). The expression unit was expressed as FPKM (frequency per kilobase million). (TIF) [file pone.0246763.s001.tif]

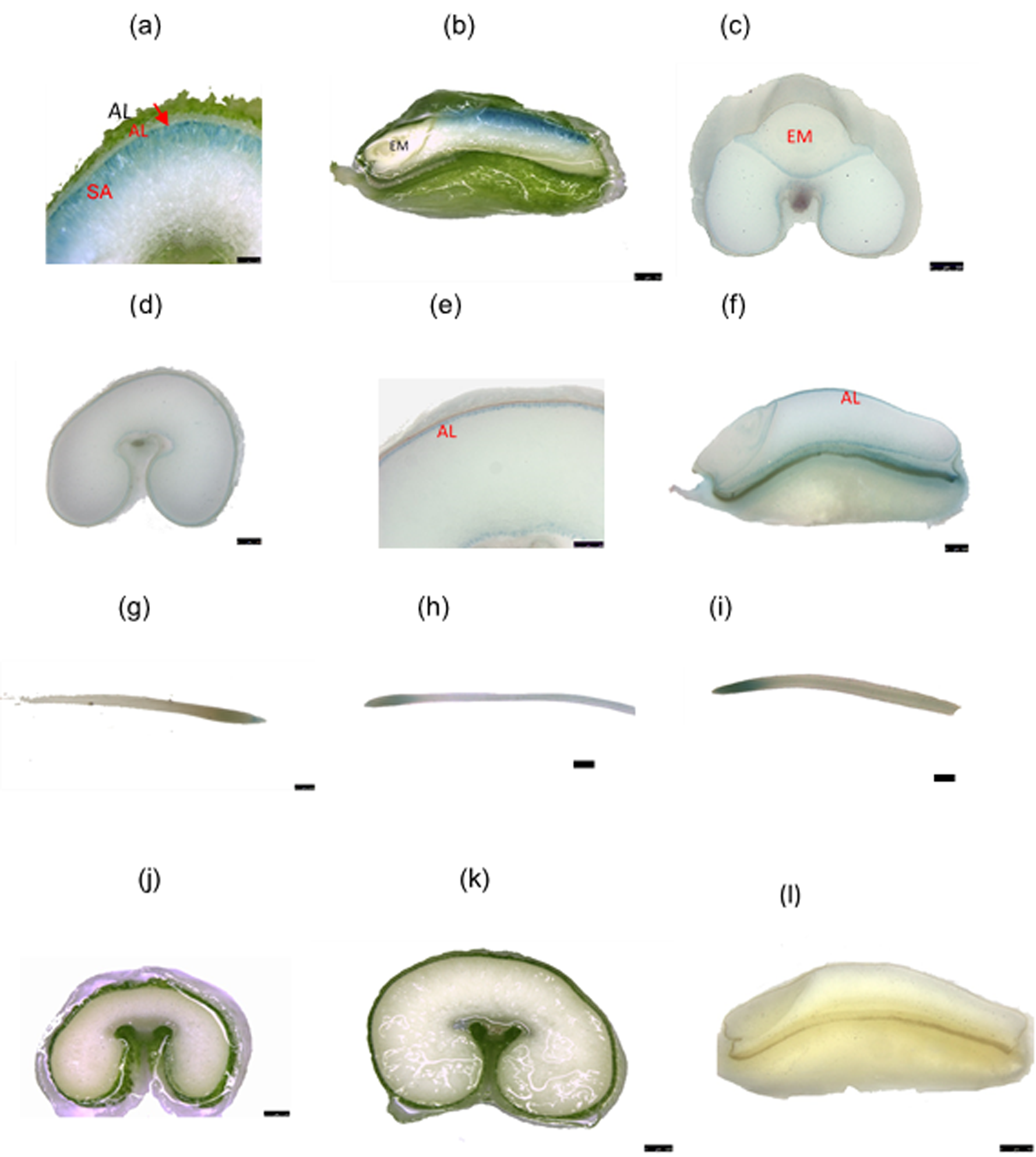

Supplement: S2 Fig — Grain GUS staining from transgenic cadenza TaAAP13D in (a-b), and TaAAP21A (c-f) at grain 21DPA (a, c, d, e), and at 28DPA (b, f). Root Gus staining from germinated grain (2–3 days) in TaAAP2B (g), TaAAP13D (h), and TaAAP21A (i). Negative control of Cadenza (j-l) at 14DPA (j), 21DPA(k), and 28DPA(l). The images were visualized using fresh grain (a, b, j, k) or after serial ethanol de-staining (c-i, l). Scale bars represent 750μm (b, l), 500μm (c, g, f, j, k), and 250μm (a, h, g, h, I). AL: Aleurone; EM: embryo; SA: sub-aleurone cells. (TIF) [file pone.0246763.s002.tif]

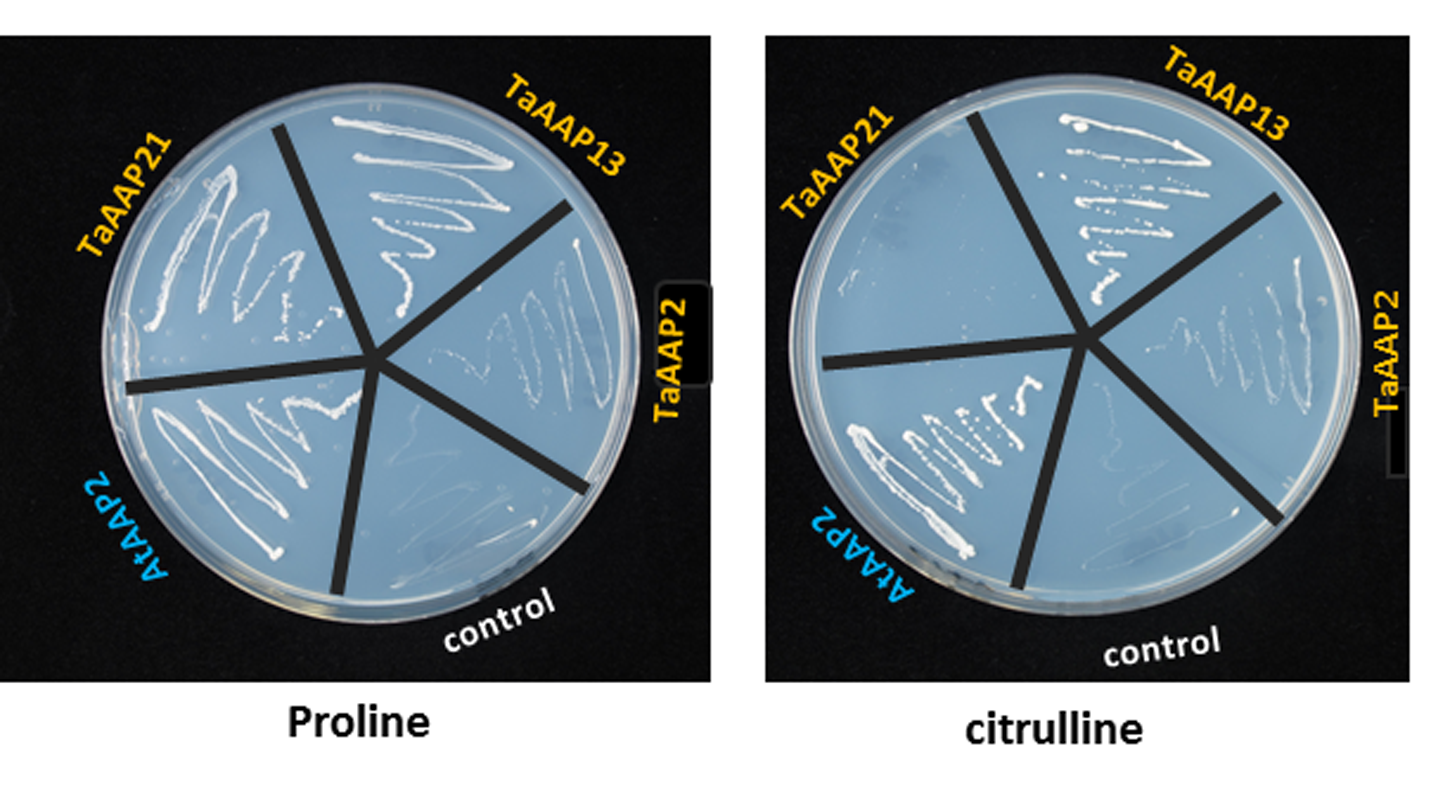

Supplement: S3 Fig — The yeast transformants with TaAAP2, TaAAP13, TaAAP21, AtAAP2 (positive control) and yeast with empty vector pDR196 (negative control) were grown on selective medium containing 1mM proline or citrulline respectively. (TIF) [file pone.0246763.s003.tif]

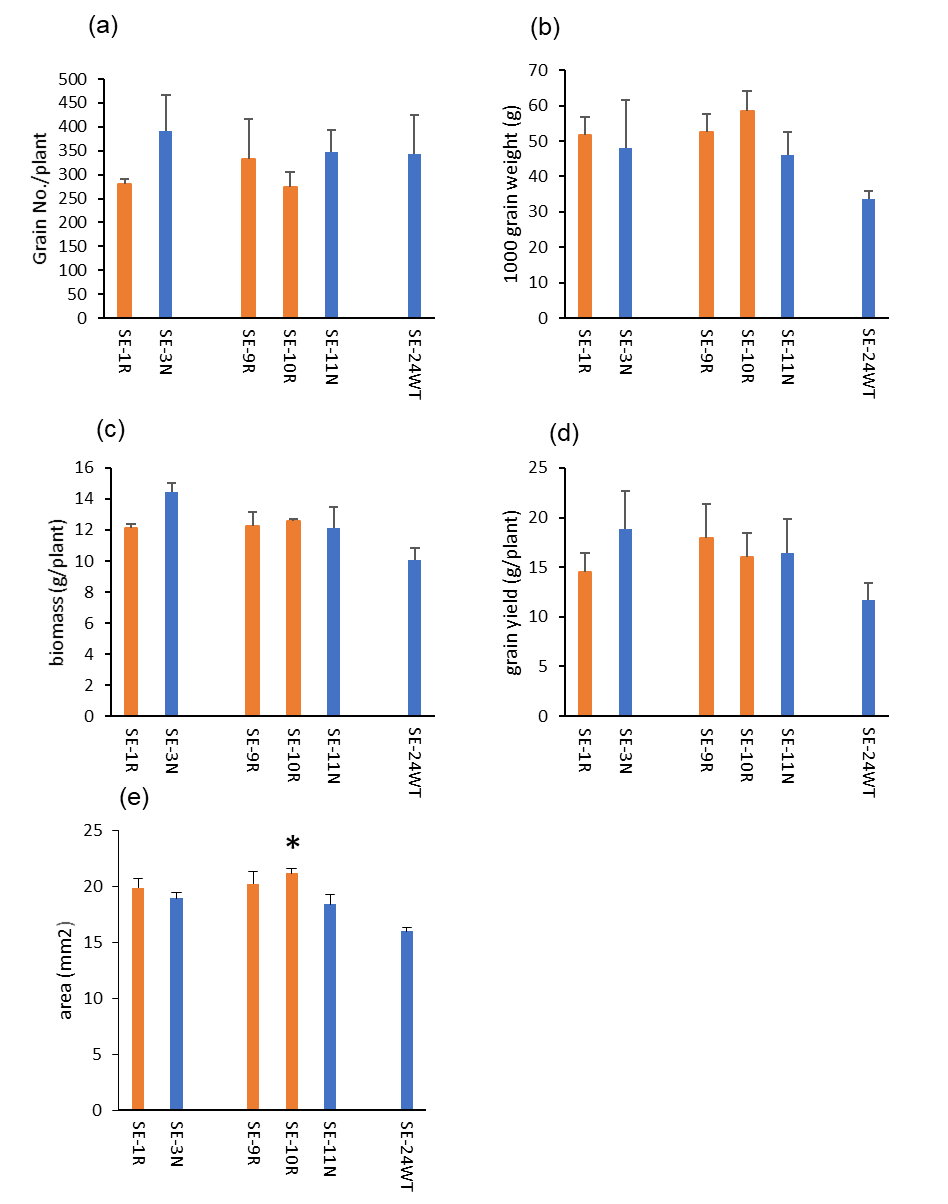

Supplement: S4 Fig — (a), Grain number per plant. (b), 1000 grain weight (g) at 15% moisture. (c), Biomass for above ground vegetative tissue (g) per plant. (d), Grain yield (g) per plant. (e), Grain areas. There is no statistically significant difference between transgenic lines and nulls except SE-10R and SE-11N in grain area. Significant differences were detected using ANOVA (P<0.05, F-test). (TIF) [file pone.0246763.s004.tif]

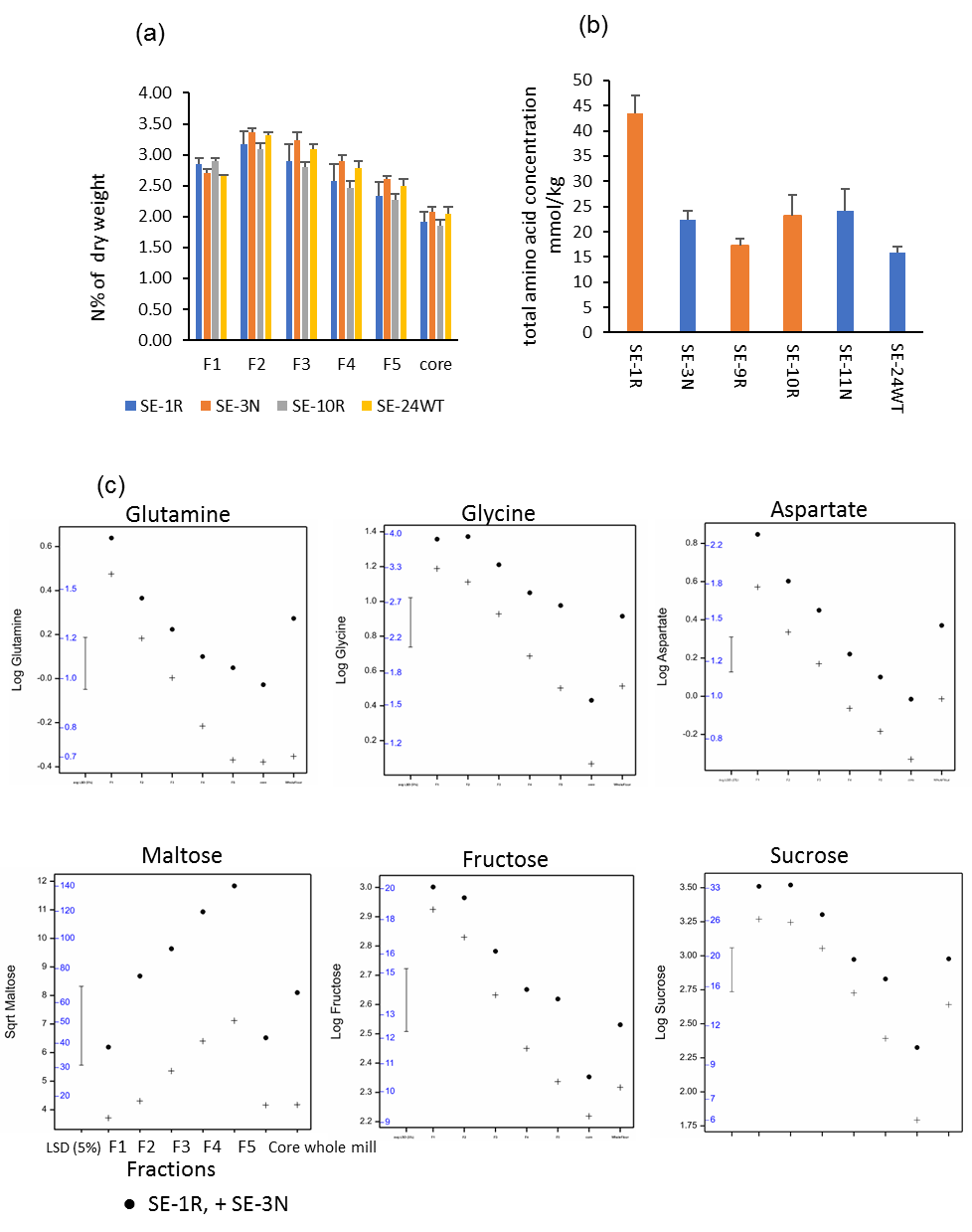

Supplement: S5 Fig — (a), Nitrogen concentration in pearling fractions (F1, F2, F3, F4, F5 and core) of the RNAi lines (SE-1R, SE-10R), Null (SE-3N), and non-transgenic line (SE-24WT). There is no statistically significant difference between SE-1 and SE-3 (P<0.05, F test). (b), Total free amino acid content in whole grain flour of RNAi lines (SE-1R, SE-9R, SE-10R), null lines (SE-3N, SE-SE11N) and non-transgenic line (SE-24WT, cadenza) determined by HPLC. (c), The metabolite changes of pearling fractions and whole grain flour by 1H-NMR in SE-1R (RNAi) and SE-3N (Null). The data on the outside and inside Y axis represent log and original amino acid concentrations (mg/g dry weight), respectively. F1, F2, F3, F4, F5 and core are mainly enriched in bran (F1), aleurone (F2), sub-aleurone (F3), towards inner endosperm (F4, F5 and core). (TIF) [file pone.0246763.s005.tif]

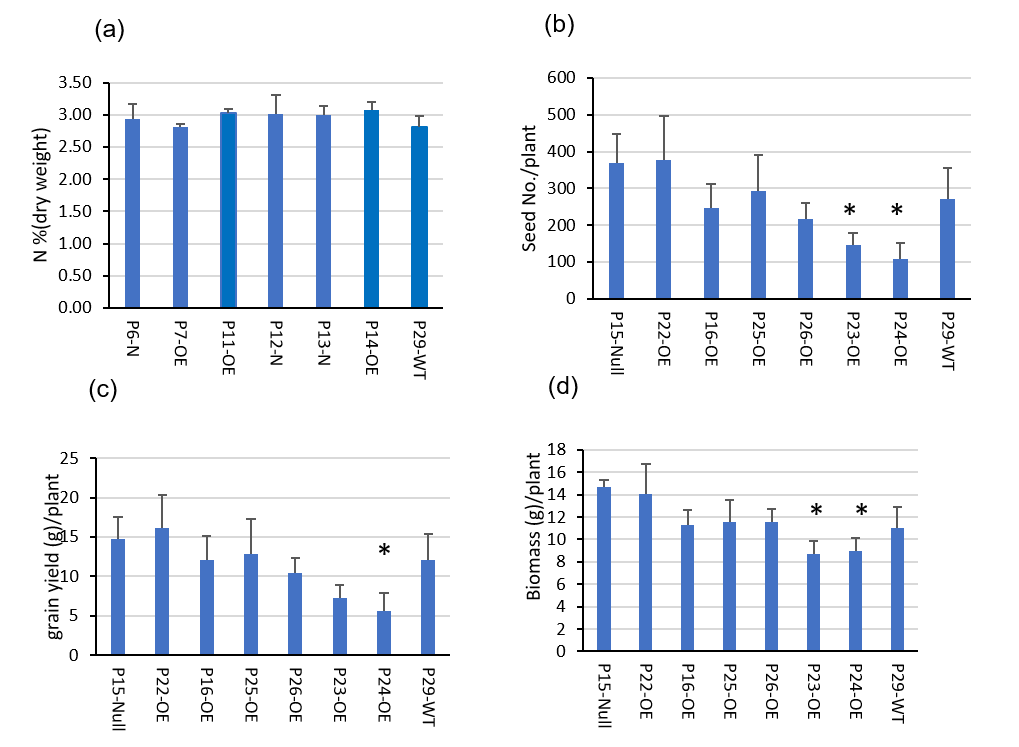

Supplement: S6 Fig — (a), wholemeal flour nitrogen concentration of overexpression of TaAAP13 lines under maize promoter Ubiquitin. (b)-(f), Overexpression of TaAAP13 lines under the control of wheat HMW-GS 1Dx5 promoter. (b), Seed number per plant. (c), Grain yield (g) per plant. (d), Biomass (g) per plant. Significant differences were detected using ANOVA (P<0.05, F-test) and were indicated with asterisk (*). (TIF) [file pone.0246763.s006.tif]

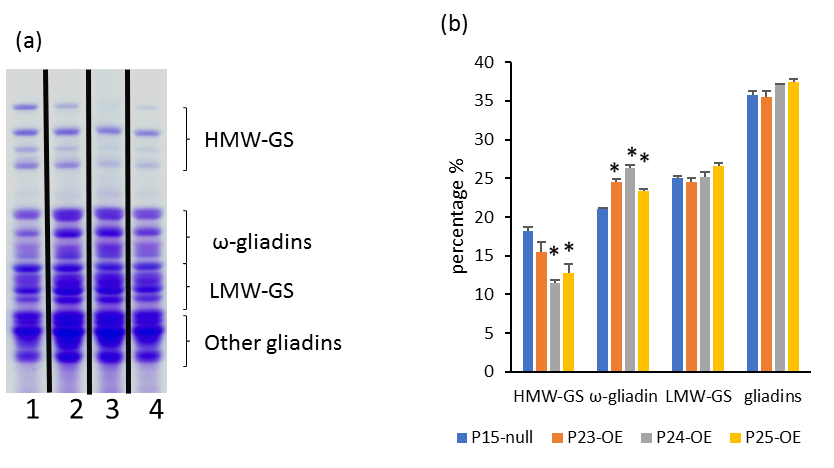

Supplement: S7 Fig — (a), SDS-PAGE of total protein, lane1: P-15null, 2: P23-OE, 3: P24-OE, 4: P25-OE. The image was spliced together from the same original gel (in S10 Fig. P15-R1, P23-R1, P24-R2, and P25-R3 respectively). (b), The protein composition of HMW-GS, ω-gliadin, LMW-GS, and other gliadins. Percentage (%) is each protein group as a % of total glutens. Significant differences were detected using ANOVA (P<0.05, F-test) and were indicated with asterisk (*). (TIF) [file pone.0246763.s007.tif]

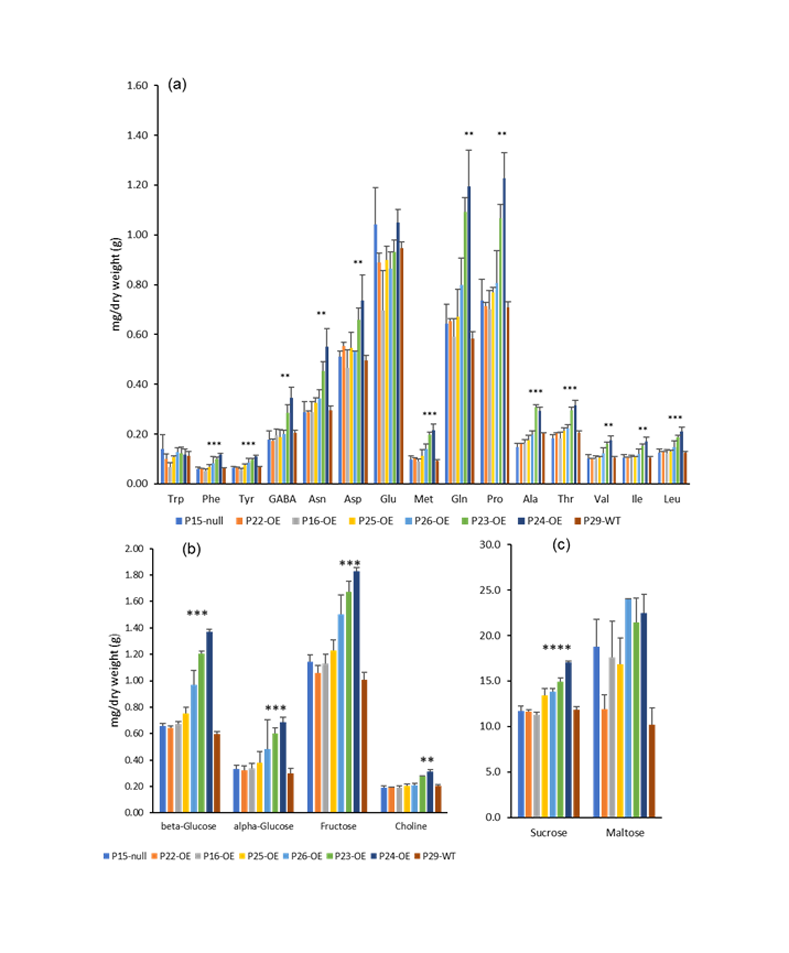

Supplement: S8 Fig — a), Free amino acids. (b-c), Other metabolites. Significant differences were detected using ANOVA (P<0.05, F-test) and were indicated with asterisk (*). (TIF) [file pone.0246763.s008.tif]

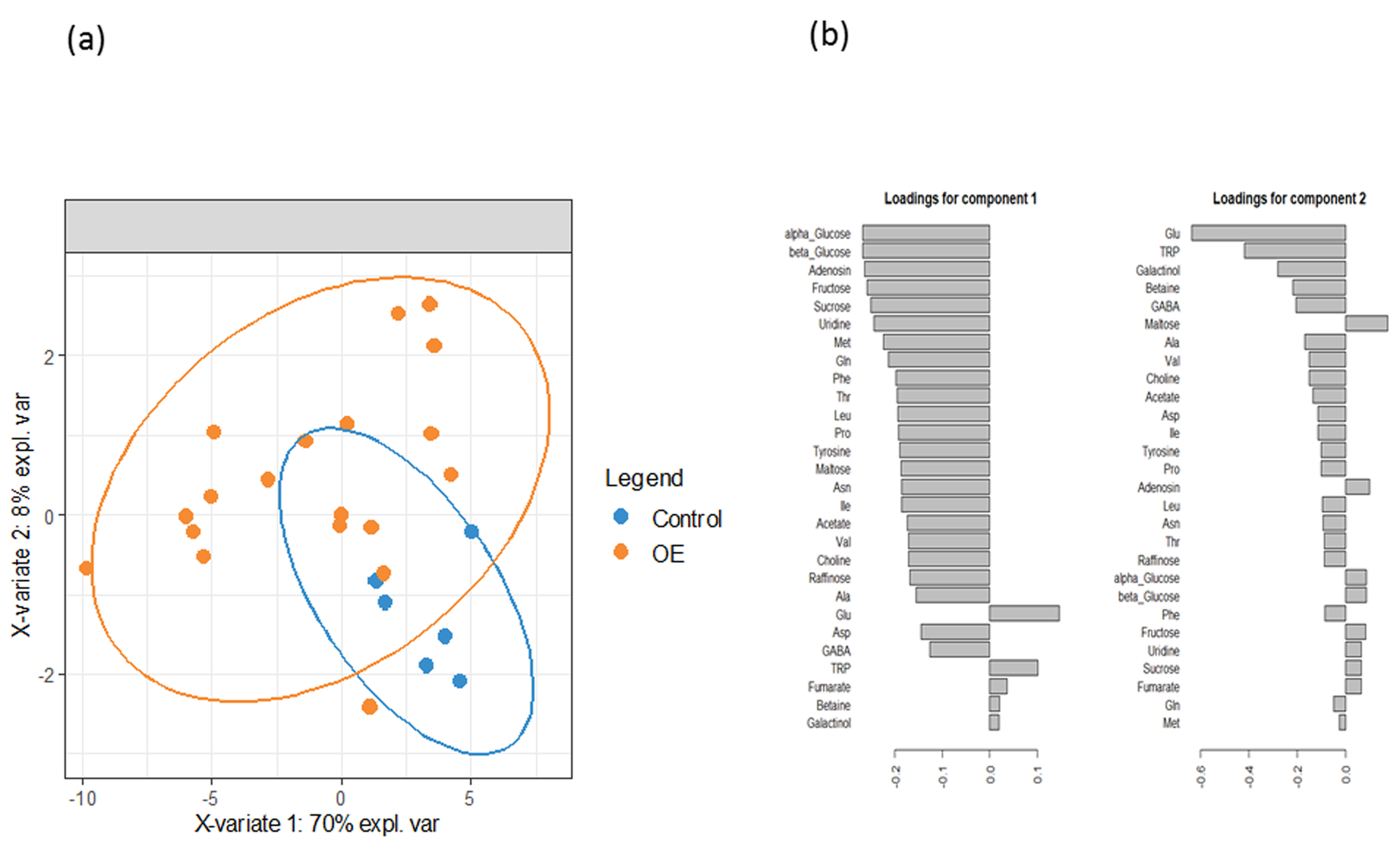

Supplement: S9 Fig — (a), PLS-DA plot. P15-null and P29-WT as control compared with all six overexpression lines (OE) (P16-OE, P22-OE, P23-OE, P24-OE, P25-OE, and P26-OE). (b), Loading of PLS-DA plot. (TIF) [file pone.0246763.s009.tif]

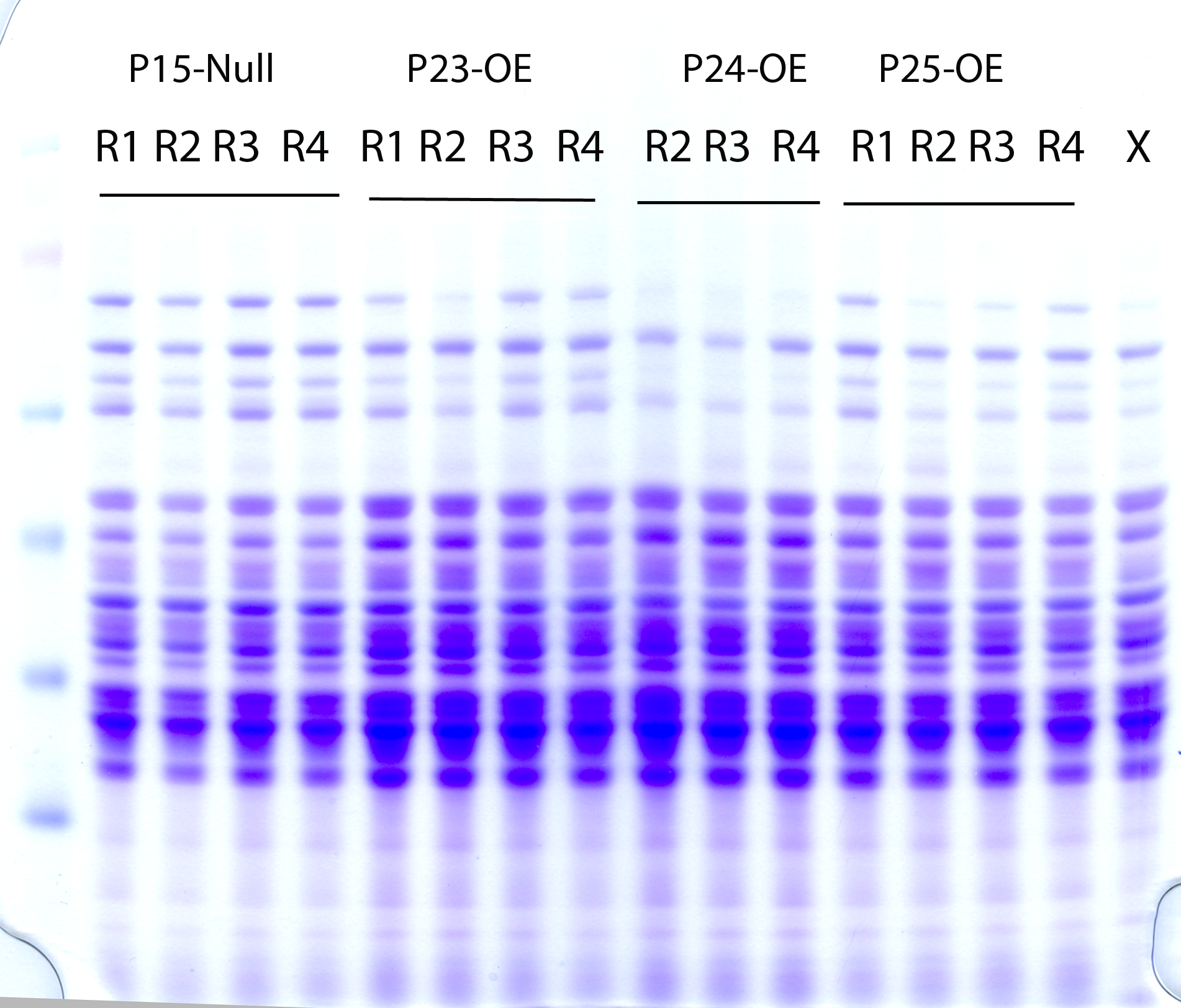

Supplement: S10 Fig — (TIF) [file pone.0246763.s010.tif]
