# Supplementary material for: Wheat amino acid transporters highly expressed in grain cells regulate amino acid accumulation in grain
Source: PLoS One. 2021 Feb 19;16(2):e0246763. doi: 10.1371/journal.pone.0246763 (PMC7894817; doi:10.1371/journal.pone.0246763)
Supplement: S1 Table — Underlined sequences were added restriction enzyme digestion sites. (DOCX) [file pone.0246763.s011.docx]

**S1 Table. Primers for RT-qPCR, promoter cloning, full length CDS cloning of *TaAAP2*, *TaAAP13*, and *TaAAP21* into yeast vector, RNAi and overexpression plasmid vector constructs.** Underlined sequences were added restriction enzyme digestion sites.

|  | Forward primer | Reverse primer |
| --- | --- | --- |
| TaAAP2B  promoter | GTC**GTTTAAAC**GGTGATGGCCGATTGAACAA | **GTCCCATGG**CGTGGGACATGGGGCGAGATGTTGCGGACTTTGT |
| TaAAP13D promoter | GTC**GTTTAAAC**ATGTCACGGCAGTATCACCA | GTC**CCATGG**CATCAGGGAAGCGATGGCAAC |
| TaAAP21A  promoter | GTC**GTTTAAAC**TGGAGGAAGAGGTGGTAGCA | GTC**CCATGG**CCCCGCCTCCG |
| TaAAP2  RNAi | CATCATGGAGATCTTCTTCTCGCA | GCTTGTACAGTCCAGCAGCCAGAAGGGCTCGTA |
| TaAAP13 RNAi | GTCAGATCTCGGGCACGGTGTGGACGGCGA | GCTTGTACACCGAGCCCGATGGTGGAGTAGGT |
| TaAAP21 RNAi | GTCAGATCTAGACGATGCGGAAGGCGACGCT | CCGGATGTACATCTCCACGGGGA |
| TaAAP2B  QPCR | TTTCCACAGACCGGGAAGTTA | CAGTGGACTCGAGTGTGTTTG |
| TaAAP13A  QPCR | \| CGGCTGAGCGATATTTGTGT \| \| --- \| \|  \| | GTTCACACAAGAAGCTCGGT |
| TaAAP13B  QPCR | \| CATCGGCGAGCTCAAGGAG \| \| --- \| \|  \| | CTCTTGCAGCACACAATCCG |
| TaAAP13D  QPCR | \| TACGGATTGTGTGCTGCATG \| \| --- \| \|  \| | ACGACGAAGAAAATGCAGGG |
| TaAAP21A  QPCR | \| TAGTCCGGGATTGAACGAGG \| \| --- \| \|  \| | TCGCACTGTCTACGATCGAA |
| Internal control QPCR (Ta54227) | CAAATACGCCATCAGGGAGAACATC | CGCTGCCGAAACCACGAGAC |
| TaAAP2 gene for yeast | \| CTCGCCCCATGTCCCACGCCATG \| \| --- \| \|  \| | TCACCCCCGGCGGAACGGGTT |
| TaAAP13 gene for yeast | \| TTCCCTGATGGAGAAGAAGCA \| \| --- \| \|  \| | AGGTTGGTAATGGTAATTTGTT |
| TaAAP21 gene for yeast | \| GCGGAGGCGGGGCCATGGACGT \| \| --- \| \|  \| | CAGGCCTTCACGACTTGGTGGTGA |
| TaAAP13  OE QPCR | CTCCAGCAACCCCTACATGA | CCGATGGTGGAGTAGGTGAA |
