# Supplementary material for: Wheat amino acid transporters highly expressed in grain cells regulate amino acid accumulation in grain
Source: PLoS One. 2021 Feb 19;16(2):e0246763. doi: 10.1371/journal.pone.0246763 (PMC7894817; doi:10.1371/journal.pone.0246763)
Supplement: S2 Table — Cross (×) represents that the AAP is unable to transport this amino acid; Tick (√) represents that the AAP is able to transport this amino acid. † indicates the TaAAP is unable to complement yeast mutant at low Arginine concentration 0.5g/l, but is weakly complement yeast mutant at high Arginine concentration at 1g/l. (DOCX) [file pone.0246763.s012.docx]

**S2 Table. The amino acids of *TaAAP2*, *TaAAP13*, and *TaAAP21* complementation in yeast mutants.** Cross (×) represents that the AAP is unable to transport this amino acid; Tick (√) represents that the AAP is able to transport this amino acid. † indicates the *TaAAP* is unable to complement yeast mutant at low Arginine concentration 0.5g/l, but is weakly complement yeast mutant at high Arginine concentration at 1g/l.

| **Amino acids** | **TaAAP2** | **TaAAP13** | **TaAAP21** | **property** |
| --- | --- | --- | --- | --- |
| **Pro** | **√** | **√** | **√** | neutral |
| **Gln** | **×** | **√** | **V** | neutral |
| **Gly** | **×** | **√** | **×** | neutral |
| **Leu** | **√** | **√** | **√** | neutral |
| **lle** | **√** | **√** | **√** | neutral |
| **Met** | **√** | **√** | **√** | neutral |
| **Phe** | **√** | **√** | **√** | neural |
| **Val** | **√** | **√** | **√** | neutral |
| **Thr** | **√** | **√** | **√** | neutral |
| **Trp** | **√** | **×** | **×** | neutral |
| **Tyr** | **√** | **√** | **√** | neutral |
| **Glu** | **×** | **√** | **√** | Acid |
| **Arg** | **×** | **√†** | **√** | Basic |
| **Lys** | **×** | **√** | **×** | basic |
| **GABA** | **×** | **×** | **×** |  |
| **Citrulline** | **√** | **√** | **×** |  |
