# Supplementary material for: Wheat amino acid transporters highly expressed in grain cells regulate amino acid accumulation in grain
Source: PLoS One. 2021 Feb 19;16(2):e0246763. doi: 10.1371/journal.pone.0246763 (PMC7894817; doi:10.1371/journal.pone.0246763)
Supplement: S4 Table — The data are expressed as means±SE (standard error). Grain size LSD (5%) = 1.72; grain length LSD (5%) = 0.22; grain width LSD (5%) = 0.22. * represents a difference exceeding the LSD 5% level compared with P15 null using ANOVA. (DOCX) [file pone.0246763.s014.docx]

**S4 Table. The grain size (area), length, and width of pHMW-TaAAP13 overexpression lines.** The data are expressed as means±SE (standard error). Grain size LSD (5%) =1.72; grain length LSD (5%)=0.22; grain width LSD(5%)=0.22. * represents a difference exceeding the LSD 5% level compared with P15 null using ANOVA.

|  | grain size(mm²) | grain Length(mm) | Grain Width(mm) |
| --- | --- | --- | --- |
| P15-null | 18.44±0.46 | 6.49±0.09 | 3.62±0.05 |
| P22-OE | 18.88±0.27 | 6.47±0.09 | 3.66±0.04 |
| P16-OE | 20.17±0.75* | 6.52±0.10 | 3.92±0.10* |
| P25-OE | 20.05±0.92 | 7.01±0.01* | 3.72±0.01 |
| P26-OE | 21.18±1.01* | 7.04±0.07* | 3.73±0.05 |
| P23-OE | 22.24±0.58* | 7.15±0.05* | 3.94±0.08* |
| P24-OE | 24.85±0.47* | 7.50±0.03* | 4.19±0.08* |
| P29-WT | 18.33±0.33 | 6.36±0.04 | 3.65±0.05 |
